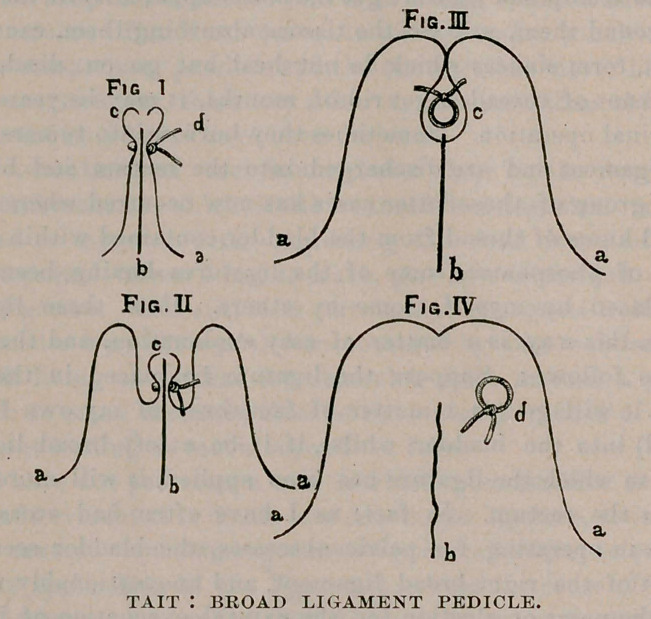# The Evolution of the Surgical Treatment of the Broad Ligament Pedicle

**Published:** 1896-07

**Authors:** Lawson Tait

**Affiliations:** Birmingham, Eng.


### BUFFALO MEDICAL JOURNAL.

VOL. XXXV. JULY, 1896. No. 12.

### Original Communications.

### THE EVOLUTION OF THE SURGICAL TREATMENT OF THE BROAD LIGAMENT PEDICLE.

By LAWSON TAIT, F. R. C. S., Birmingham, Eng.

A VOLUME which I published in 1891 was devoted mainly to the establishment of severe operative procedures for the relief of diseases of the pelvis and abdomen, especially in women. It dealt chiefly with the leading features of these proceedings, in the direction of reducing their primary mortality and establishing them on the basis of giving a reasonable return for the risks run. This, in every instance, has now been fully accomplished, and if I had confined myself to such themes in my first volume, my second would have been ready long ago; but the appearance of the first effort toward the latter has been delayed till today, for I have found so much to reconsider in the second division of my task that I have had to decide to wait, over and over again, till the effort seemed to me hopeless, and I felt at last that I should leave it to other hands to accomplish. In the intervening six years the field of surgical literature has been flooded with contributions to abdominal surgery, some good, a few very good, very many indifferent and the great bulk hardly worth notice. I have waited to see if any one would take up what seems to me the logical method of advance; but I have found none and the effort which I now begin today to produce my second volume and to indicate the lines on which I have improved and still hope to improve the second as results of our operations, may turn out to be a failure as great as any which I have just deprecated; if, however, it have anything like the success which my first volume has met with I shall be pleased indeed, and in my own opinion the second will greatly transcend the first, alike in interest and importance.

As the whole history of abdominal surgery, in the slow and tardy evolution of its first stages, turned on the treatment of the broad ligament pedicle, so my first efforts in the *secondary* evolution necessarily start at the same point ; and it is, to say the least of it, disappointing to see how little the importance of this subject has taken hold of the men who have written most fluently on abdominal surgery and who have brought out the most popular text-books. Mr. Greig Smith's book, for instance, will, to its ordinary reader, seem sufficiently imbued with my own teaching, yet he says concerning the treatment of the pedicle : "It has always seemed to me that this *quæstro vexata* of ovariotomy has been unduly magnified in its importance. To a surgeon accustomed to deal with amputated limbs the pedicle is, comparatively, a small matter." If this is the view generally of surgeons accustomed to deal with amputated limbs I hope fervently that no one in whom I have any personal interest will ever fall into their hands for the treatment of an ovarian tumor. Coming from the pen of a man like Greig Smith it can only mean that whilst he has written freely and with confidence, he has not seen enough ovariotomies to know how wholly different their pedicles can be, and that his comparison of them with the symmetrical and constant incidents of amputated limbs is the most perfect nonsense.

Similarly, the experience of one or two cases where chronically overdistended bladders had been tapped for ovarian cysts or even attempts made to remove them by abdominal sections, will some day cause him to repent bitterly of the following sentences : "I think that the advantages of catheterism before operation are somewhat exaggerated. I am convinced that it is unnecessary, and I have never done it." From these illustrations I am persuaded that the creation of abdominal surgery into a special branch of surgery was a wise movement, on the same ground that the development of ophthalmic surgery has upset the advice we used to find in a few chapters on diseases of the eye, oddly enough placed at the end of text-books on practice of medicine, that in all cases of squint the muscles supposed to be at fault were to be freely divided.

It may be useful on one other point to continue my criticism of Mr. Greig Smith's text-book, and that is his expression of opinion that "the operation of Houston, of Glasgow (1701), was almost certainly not a complete ovariotomy." But apart from the very conclusive fact that the patient lived afterwards in perfect health for thirteen years—not known in my own experience of incomplete ovariotomies—Mr. Greig Smith only lays himself open again to the charge of a defective experience. I could not understand why Houston should describe his proceedings with such photographic fidelity of detail and say nothing about his treatment of the pedicle, and I assumed that he must have divided and tied it and forgotten to mention the fact. I did not attach sufficient importance to the fact that Houston had met with one of those singular eccentricities of ovarian growth, of which my own long list was interspersed with a goodly number, and it was not till I began to dig them out and carefully investigate their details that I saw the probability of Houston's description being perfect, even in the absence of allusion to the pedicle and its treatment. The clue is in his words:

> I took a strong fir splinter, such as the poor in that country use to burn instead of candles; I wrapped about the end of this splinter some loose lint and thrust it into the wound, and by turning and twining and winding it I drew out about two yards in length of a substance, thicker than any jelly, or rather like glue first made, and hung out to dry. Its breadth was above ten inches; this was followed by . . . several large pieces of membrane, which seemed to be parts of the distended ovary. I then squeezed out all I could and stitched up the wound, and the like.

When I got a group of my own cases together it was easy to see that Houston's belonged to them and that they were a group by themselves, having peculiarities of their own, not the least curious of which is the fact that they probably present the examples, which occur beyond all doubt, of ovarian tumors undergoing spontaneous cure—examples, however, so rare that they may be entirely disregarded in any discussion of treatment.

These cases have all the same character and no description can be better than that of Houston's own experience. The cyst walls are always thin and so far as my memory and my notes serve me the tumors are sessile. In three well-marked and entirely similar cases the cyst walls had given way and the gluey contents were diffused throughout the abdomen. The cyst walls, or rather their remains, were isolated by washing out and were then found to be mere shreds, patches and strings of membrane with attached masses of cystic proliferation, by which only their inner surfaces could be recognised. These shreds and patches were quite rotten and came away from their very vague attachment in the pelvis on slight touch and no ligatures were necessary. In all three, drainage-tubes were left in at the close of the operation and large quantities of the gluey material, more or less in solution, were discharged for some days.

My belief is that in none of these three cases was the operation really necessary. Had I left them alone the gluey material would have been slowly absorbed and the shreds and patches would have contracted and disappeared, unless they had developed malignant tendencies, and there is a suspicion that this did happen afterward in one of my cases. This is a secondary termination from the risk of which no ovariotomy is free.

These three cases are, therefore, practically on a level with Houston's, only that I did a little more scientifically and with better tools what he did effectually with a bit of stick. What is the explanation of them? That question I can only answer by speculation. True pedicle they have none and it seems to me as if and at a certain point the cyst contents have a digestive power over the cyst walls and that in time the peritoneal surfaces digest the cyst contents. It may be that when we come to the base of the tumor and pull away the rotten remains, we complete the process by a rough enucleation, but I really cannot say. I saw the base of attachment of these three tumors and Houston did not see his, therefore he said nothing about it. But I saw mine with the eyes of the blind and only at my next experience will my eyes be open enough to get a true record of the state of matters. Meantime I have said of Houston that the earliest known treatment of the ovarian pedicle was probably enucleation.

Houston's operation makes a most interesting and picturesque addition to the early history of abdominal surgery, but it had no value at all, although it was in the stately transactions of the Royal Society of London, read then as now with avidity all over the world. He found no imitators.

The first really deliberate and systematic attempt to do serious operations for abdominal disease was that of Ephraim McDowell. It was, of course, received by the only argument with which it could be met, that constant refuge of human imbecility, that McDowell was a liar, and the historical fact is on record that an editor of a medical journal who spread the libel had to eat dirt and "beg pardon of God and Dr. McDowell." Nowadays there seems to be no such fear of the Deity amongst medical editors, but some day one of them will have to make similar apologies.

McDowell's method of dealing with the pedicle was what he had learned from John Bell in Edinburgh, and which was applied to all arteries and to everything containing an artery down to my own time—the long ligature. In a stump the arteries were tied with a hempen ligature, one end was cut short and the other left long, the series to decorate the suppurating stump for many days, the amusement of the house-surgeon and the terror of the patient being their removal by tugging as soon as they would come. Often they never came. How it did not strike someone to try how they would do if both ends were cut short I cannot imagine. But the difficulty set Simpson's ingenious mind to work with his visionary acupressure and he unconsciously cleared out the long ligature. How the short ligature came into use in general surgery I do not know, but in ovariotomy it appeared early in the history, but, most unfortunately, only for a short time.

In 1822, Nathan Smith, of New Haven, Connecticut, did his first case with the short ligature, dropping the pedicle in and fol-lowed this by a series of cases so successful that it must ever be the amazement of future historians of surgery how his example was not at once followed. His lesson was far more valuable than McDowell's and his action more philosophical, for he argued out the necessity for the short ligature and he used animal ligatures (pieces of kid gloves) in the belief that they would be absorbed—a conclusion most abundantly proved to be correct. His lessons were neglected, I suppose, on the ground that he also was set down as a liar. At any rate, he must have been so regarded in this country, for his statements were widely published here, yet the long ligature held sway till the advent of Baker Brown, in 1851. He in turn introduced the treatment of the pedicle by the cautery, gaining his idea doubtless from veterinary surgery.

A most important incident occurred soon after this by the invention and introduction of the clamp treatment of the broad ligament pedicle—an incident, according to his own authority, to be laid at the guilty door of Mr. Jonathan Hutchinson.

The short ligature was reintroduced and reëstablished almost simultaneously in 1878 by Bantock and myself, and has since remained the only method dealing with the broad ligament pedicle, except the cautery and clamp used by Keith to the last. Up to that time we were all astray except Keith, as we used Hutchinson's clamp with varying mortalities, running between the dreadful limits of 25 per cent. and 50 per cent. Keith alone, adhering to Baker Brown's method, brought down his mortality and swept the world with his brilliant success. Very soon, however, Keith's record was beaten by the ligature and then it became manifest that the main principle to be followed for success was that of the intraperitoneal replacement of the divided and secured pedicle and that there was little to choose so far as promising results were concerned between the cautery and the short silk ligature.

As I watched Keith operate in 1880-81 I became convinced that the cautery was the ideal method of pedicle treatment, but for the tremendous time it occupied and the large amount of physical exertion involved. Keith and I, in a half-jesting fashion, agreed to stick each to his method and after a long series of cases to compare results. But Keith ceased to publish under conditions which made comparison possible and the contrast has never been made. I do not, however, think any other conclusions than those I have indicated could ever have been derived.

I have, however, been haunted ever since by the clear, thin line of parchment to which Keith reduced his pedicles, and I have looked at my own often regretting the dead lump of lost and decomposable tissue I had to leave, girt by a moist and decomposable ligature. But still it is certain that the great bulk of these difficulties were successfully encountered by that wonderful power which living tissue has for removing dead matter, a power for which so many ravenous theories have been advanced of late years without leading us much beyond the facts.

I have not yet seen a case in which post mortem examination made it evident that death was due to the ligature, though I have seen several—some amongst my own cases I regret to say—where faulty application of the ligature has been the cause of death. If the ligature is so applied that it controls hemorrhage completely, the ligature does not contribute in any way, so far as I know, directly or indirectly to death. But it has a most unfortunate way of contributing to secondary failure, to incomplete cure and to persistent and vexatious interference with convalescence.

The first of the instances of this kind as being never fatal, but as being the more common and more alarming of the two to be mentioned, is the occurrence of broad ligament hematocele. Speaking to Keith about this, his answer impressed me with the belief that the accident did not occur in his practice, and he was far too shrewd and anxious an observer to miss it if it had occurred frequently. The users of the clamp certainly did not place its occurrence on record, but with a mortality of 25 per cent. raging round them it is not likely that they would observe it if it did occur, and when it occurred I think death must have been inevitable. I cannot imagine any more certainly fatal combination than a broad ligament hematocele and a stinking stump. But still the question stares us in the face, Does the ligature cause or facilitate the occurrence of broad ligament effusion ? I confess I strongly suspect that it does, and this is one of the reasons of my present paper.

The second incident which tells against the ligature is the occurrence of a number of cases, 3 or 4 per cent., in which the garrotted stump and ligature get the better apparently of the living tissue around them, prevent the tissues absorbing them, cause suppuration, form sinuses which do not heal but go on discharging till the knot of thread is got rid of, months, it may be years, after the original operation. Sometimes they burrow into tumors of the broad ligament and are discharged into the rectum and bladder. Quite a group of these latter cases has now occurred where I have removed knots of thread from the bladder contained within a thick deposit of phosphates, some of the ligatures having been originally placed by myself, some by others. How these ligatures travel in this way is a matter of easy explanation, and that is, I think, as follows : Suppose the ligature be placed in the right pedicle, it will go (as a matter of fact three of my own have so traveled) into the bladder, whilst, if it be a left broad ligament pedicle to which the ligature has been applied, it will more likely get into the rectum. In fact, as I have often had occasion to observe in operating for pelvic abscesses, the bladder seems the road out of the right broad ligament, and unquestionably the rectum is the point of election for the natural evacuation of the left Parametric cavity.

The method of exclusion of the ligature is curious and interesting, for it seems difficult to imagine at first sight that a ligature tied inside of the peritoneum would, as a matter of course, under certain conditions, work its way through that membrane and into an extra-peritoneal district.

Its subsequent journey is a matter of notoriety and of long and protracted suffering for the patient. I could, unfortunately, point to a number of my patients who have for months, and even years, been undergoing this torture from what I call a " dead " ligature. I have made various attempts to relieve them, some, I am glad to say, successfully and others, I regret to say, with failure.

But they all got well in time and none, so far as I know, have died in the process.

It happens thus: The ligature (Fig. I., *d*) is tethered to the floor of the pelvis by the vessels and nerves it embraces. The peritoneum (*a*) is raised up and round it and finally over it by the effusion of blood, until a tube of inverted peritoneum (*c*) is made with the ligature at the base (Figs. II. and III.) The mouth of this inverted tube is closed by adhesion at its intraperitoneal end and opened at the ligature end by the subsequent suppuration, and the loosened ligature drops into the increasing and purulent cavity of the broad ligament (Fig. IV., *b*).

TAIT: BROAD LIGAMENT PEDICLE.

For these two reasons I want to get away from the ligature if I can, and after years of experimenting and thought I think I have at last succeeded. But the difficulties have been so great that I have not yet ventured to apply my plan to a living subject. Fortunately, however, for my consistency, dead animal tissue is helping me thoroughly and experiments on animals are unnecessary, for this purpose at least.

My constant feeling has been that in the cautery we should find the solution of this difficulty, and it only shows how careful we ought to be in all our experiments in surgery, that it was, and not till after at least two years' consideration of the subject that it dawned on me that we did not understand how the cautery really works. I do not think that either Baker Brown or Keith understood how they arrived at their certain and magnificent results. I am sure the bystanders did not. Everyone whom I have asked questions on the subject has answered, "Oh, yes, sear the stump; barbarous practice, going back to the days before Ambrose Parè." But Keith didn't sear the stump. It is true he burnt a piece of it off after securing it with Baker Brown's clamp, and if searing had been the means of his success he would have stopped there. But he went on for about twenty minutes or half an hour, rubbing the clamp with his cautery and cleaning it with towel or sponge, until the onlookers got weary of this proceeding and thought Keith was finical. What he had done was really this, and I found it out only after much experimenting, that he had seized a transverse strip of the pedicle between the iron blades of his clamp, screwed the blades up tight and then heated his blades up to cooking point (that is, practically, between 180° and 190° F.) and carefully maintained that temperature till the enclosed strip of pedicle was cooked dry into a strip like parchment. Now, Keith either did not understand what he was doing, but acted merely by rule of thumb, or he kept his real reason for the cautery a profound secret. Had he seared the tissue the probability either is that hemorrhage would have occurred soon after the patient recovered from shock, or that the burnt areas would have caused pelvic suppuration—it would not have been absorbed.

Having satisfied myself of the validity of this conclusion, I set to work to contrive a better method of arriving at the same results and after many failures I have found it in the method of cautery by electricity. Knowing the resistance of a certain piece of platinum wire, I embedded it in a box of silver and isolated it by means of plaster-of-Paris or Kaolin. Two such boxes are placed face to face and connected by necessary binding screws or in the blades of a pair of clamp forceps. The boxes are placed opposite each other on a pedicle and are screwed together. An electric current of known strength and under the control of a rheostat is now turned on to heat the boxes, or better still the boxes are heated first and then applied to the pedicle, as this method saves time. The boxes are, of course, enclosed in ivory, or other bad conductor of heat, to save alike the heat and the tissues for which the heat is not wasted, or are well packed by sponges. The apparatus I now show will so cook an ordinary pedicle that hemorrhage will be impossible and suppuration unlikely in the extremest degree, in a space of about six to eight minutes. The same principle has been applied to instruments for the arrest of parietal and omental hemorrhage and for the simplification of the operation of total removal of the uterus, also for operating on hemorrhoids. I do not advocate my proposals as likely to reduce the primary mortality of operations. I believe that has been done so far as may humanly be possible. But I am quite sure that they and some others on similar lines will go far to relieve our secondary results, being more satisfactory and encouraging alike to our patients, ourselves and to the art of surgery.

BIRMINGHAM, June, 1896.

## Figures and Tables

**Fig I; Fig. II; Fig. III; Fig. IV f1:**